# Spontaneous Intracranial Hypotension in Pregnancy with Aggravated Comorbidity: A Case Report and Review of Diagnostic and Management Challenges

**DOI:** 10.3390/reports8040231

**Published:** 2025-11-11

**Authors:** Taruna Agrawal, Jhia Jiat Teh, Konstantinos S. Kechagias, Zak Jefferson-Pillai, Kanwaljeet Kaur Sandhu, Sarah-Jane Lam

**Affiliations:** 1 Department of Obstetrics and Gynaecology, The Hillingdon Hospitals NHS Foundation Trust, London UB8 3NN, UK; jhia.teh@nhs.net (J.J.T.); konstantinos.kechagias1@nhs.net (K.S.K.); z.jefferson-pillai@nhs.net (Z.J.-P.); kanwaljeet.sandhu3@nhs.net (K.K.S.); sarah-jane.lam@nhs.net (S.-J.L.); 2 Department of Metabolism, Digestion and Reproduction, Imperial College London, London SW7 2AZ, UK

**Keywords:** spontaneous intracranial hypotension, epidural blood patch, pregnancy, gestation, antenatal

## Abstract

**Background and Clinical Significance:** Spontaneous intracranial hypotension (SIH) is a rare cause of headache characterised by cerebrospinal fluid (CSF) leakage, with an estimated incidence of 3.7 to 5 cases per 100,000 per year, peaking around the age of 40 years. Its diagnosis and management are particularly challenging in pregnancy due to overlapping symptoms and limited diagnostic options. **Case Presentation:** We report the case of a 42-year-old pregnant woman at 14 weeks of gestation presenting with a history of orthostatic headache and facial sinus tenderness, later diagnosed as spontaneous intracranial hypotension. **Conclusions:** Headache is a common clinical symptom that may be associated with a wide spectrum of underlying conditions, ranging from benign causes such as migraine or tension-type headache to potentially life-threatening pathologies, including subarachnoid haemorrhage. This case illustrates the diagnostic complexity of SIH in pregnancy and the importance of a multidisciplinary approach and vigilance for neurological symptoms during pregnancy.

## 1. Introduction and Clinical Significance

Spontaneous intracranial hypotension (SIH) is a rare neurological condition characterised by cerebrospinal fluid (CSF) hypovolemia in the absence of preceding dural puncture or trauma. It has an estimated incidence of 3.7 to 5 cases per 100,000 per year, though likely underdiagnosed due to the heterogeneity of clinical presentation and the associated diagnostic challenges [1]. It is typically characterised by an orthostatic headache, which worsens in the upright position and improves when in a supine position, and is often accompanied by nausea, neck stiffness, tinnitus, or diplopia [1]. The condition is usually caused by spontaneous CSF leaks along the spinal axis, often due to structural dural weakness or meningeal diverticula [2]. Additionally, individuals with connective tissue disorders, such as Marfan syndrome and Ehlers–Danlos syndrome, may be predisposed to spontaneous CSF leaks due to inherent dural weaknesses associated with these conditions [3]. Spontaneous CSF leaks are rare, most frequently arising in the thoracic spine, with the cervicothoracic junction being the second most common site.

While SIH can occur in any population, the peak incidence is around 40 years of age, and the condition affects women more frequently than men, with a female-to-male ratio of approximately 2:1 [1]. Nevertheless, SIH during pregnancy and the puerperium is particularly rare and underreported in the literature. In pregnancy, severe, quick-onset headaches raise concern for life-threatening conditions such as preeclampsia, cerebral venous sinus thrombosis (CVST), and subarachnoid haemorrhage, often overshadowing rarer diagnoses such as SIH [4]. Given the rarity of SIH in pregnancy and the unknown incidence in pregnancy, evidence is largely limited to case reports and case series, with no consensus on the optimal management for these patients [5]. In this article, we present a case of SIH in pregnancy requiring multidisciplinary input and serial neuroimaging for diagnosis and management.

## 2. Case Presentation

A 42-year-old White British woman, gravida 5 para 1 + 3, with a body mass index (BMI) of 43 kg/m^2^, venous thromboembolism (VTE) risk score of 3, presented at 13 weeks of gestation with a sudden-onset, thunderclap headache localised to the forehead, radiating to the occiput and the cervical spine. The headache worsened significantly upon standing, with a verbal rating scale (VRS) of 10/10 and improved when lying down, with a VRS of 5/10 indicating an orthostatic component. The headache was not relieved by regular analgesics such as paracetamol (1 g four times a day) and codeine (30 mg four times a day). There was no history of fever, recurrent headaches, migraines, essential hypertension, or head trauma. The patient reported having mild nasal congestion. On examination, there were no focal neurological deficits, neck stiffness, or photophobia. Facial sinus tenderness was noted on examination.

Her past medical history included epilepsy (on lamotrigine 75 mg twice daily), hypothyroidism (on 100 mcg levothyroxine), and rheumatoid arthritis with Sjögren’s syndrome overlap (on hydroxychloroquine 200 mg twice daily), with positive anti-Ro antibodies. She was started on aspirin 150 mg at her 12-week antenatal appointment for prevention of foetal growth restriction, due to her previous baby being on the 3rd centile.

She was admitted under the medical team. An initial CT head performed at 14 weeks’ gestation revealed chronic bilateral maxillary sinusitis with mucosal thickening and bony remodelling. She was treated for acute sinusitis with antibiotics. ENT opinion was sought, and the patient was advised to use nasal irrigation with steroids. However, there was no improvement in the headache. Two days later, a lumbar puncture was attempted; however, the attempt was unsuccessful. An ophthalmology review was undertaken simultaneously to rule out uveitis, which was unremarkable.

A neurology opinion was taken, and she was planned for a repeat lumbar puncture and further imaging of the head. Laboratory tests were performed to rule out any inflammatory, viral, bacterial, fungal, tuberculosis, immunodeficiency, autoimmune, or infectious pathology. Lumbar puncture was successful on the second attempt at 16 weeks’ gestation. The opening pressure could not be measured as it was performed in a sitting position; however, low flow of CSF was observed. CSF was sent for culture and sensitivity, oligoclonal bands, beta D-glucan to rule out any autoimmune, bacterial, viral, or fungal meningitis. CSF also showed the presence of lymphocytic pleocytosis, a characteristic of aseptic meningitis. Given the negative CT head and no history of a traumatic lumbar puncture (except the unsuccessful first LP attempt), she was then treated for aseptic meningitis empirically. Despite that, there was still no improvement in the headache. Given the wide differential diagnoses, imaging including MRI head without contrast, MR Venogram, and CT angiogram head were arranged to rule out any other intracranial pathology such as reversible cerebrovascular vasoconstriction syndrome (RCVS), subarachnoid haemorrhage, cerebral venous thrombosis, and intracranial aneurysm. An MRI of the head revealed meningeal thickening overlying both cerebral hemispheres, rather than a subdural haemorrhage, and no hydrocephalus. MR venography excluded cerebral venous sinus thrombosis. CT angiography ruled out intracranial arterial aneurysm and reversible cerebral vasoconstriction syndrome (RCVS).

**Figure 1:**
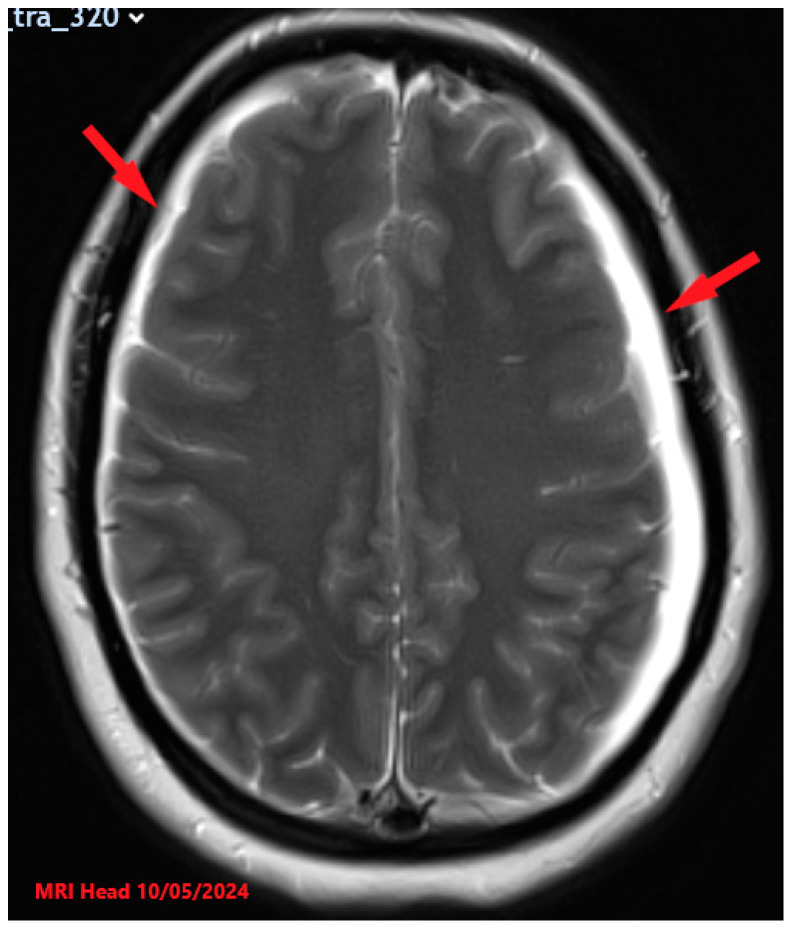
T2-weighted MRI Head with contrast axial view image findings showing initial presentation with significant bilateral subdural CSF collections (red arrows) and associated pachymeningeal enhancement and thickening.

**Figure 2:**
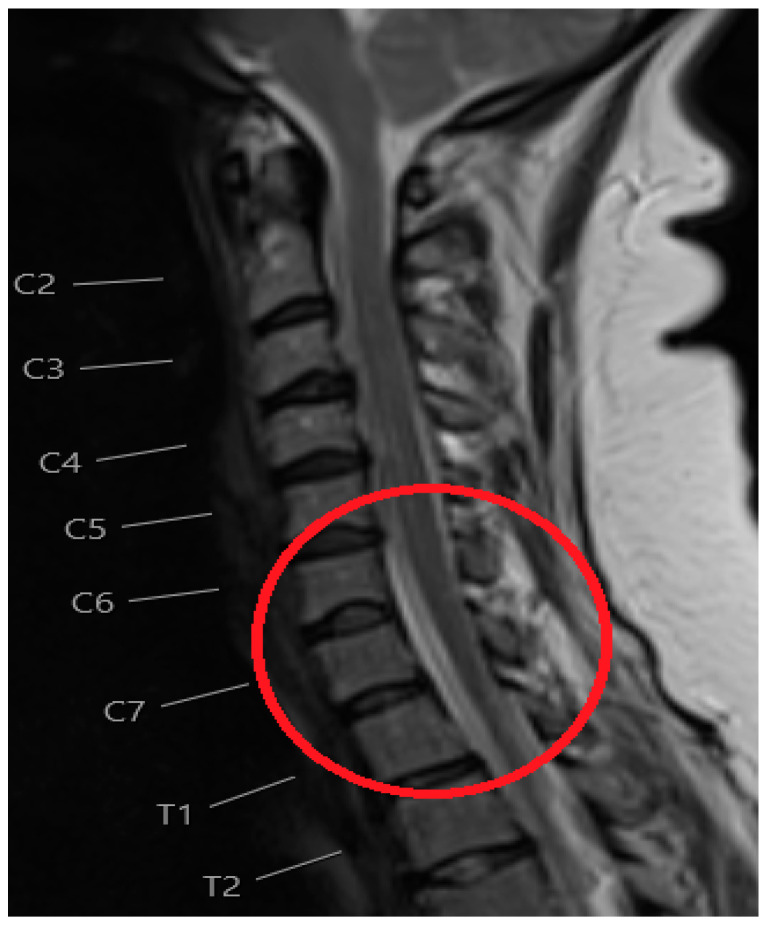
T2-weight MRI Spine sagittal view image shows minimal CSF in the basal cisterns and cerebellar tonsils noted to be lower lying extending beyond the foramen magnum (brain sagging). Persistent extradural CSF is seen within the cervical and thoracic spine, best seen posterior to the C7 vertebral body as circled in red.

She was then assessed by the local neurology team, including neurologists and neurosurgeons, who suspected low-pressure headaches and the possibility of a spontaneous leak. The local neurology multidisciplinary team recommended a repeat MRI head, spine, and orbits at 16 weeks and 3 days of gestation. MRI head, spine, and orbits demonstrated bilateral shallow subdural collections and extradural fluid within the spinal canal, meningeal thickening, and venous engorgement (Figure 1 and Figure 2). The case was discussed in a tertiary-level multidisciplinary team meeting for neuroradiology. A diagnosis of spontaneous intracranial hypotension was established based on the clinical presentation of a thunderclap headache with prominent postural features that worsened on standing, in conjunction with MRI findings of meningeal thickening. This was further supported by lumbar puncture results, which demonstrated xanthochromia and lymphocytic pleocytosis. She was then managed on the lines of treatment for spontaneous intracranial hypotension in pregnancy. She was initially managed with conservative management, including prolonged bed rest, adequate hydration, and avoidance of any strenuous activities such as bending, straining, with the aim of keeping the pain score below 4/10. She was started on prophylactic low molecular weight heparin to prevent the risk of venous thromboembolism in pregnancy. There was still no significant improvement in the headache with regular paracetamol and codeine. Her neurological examination as inpatient had always been unremarkable with no focal neurology.

Her case was discussed with a tertiary centre obstetric neurologist regarding the safety of epidural blood patches in pregnancy. She subsequently had a blind epidural blood patch (EBP) at 16 weeks and 4 days of gestation. 40 mL of blood was injected at L3/4 level until the patient experienced discomfort and hence the procedure was stopped. This provided initial relief with headache, particularly with the resolution of pressure behind the eyes and pain when looking upwards. However, the headache recurred and worsened over time to an intensity of 9/10. An interval MRI of the head and cervical spine showed that the low-pressure features from the previous scan had deteriorated, with a slight increase in subdural collections. She was then discussed again at the neurology MDT and subsequently had a second blind EBP at 18 weeks and 2 days of gestation. It was performed at level of L3/4 with 21 mL of blood until the patient reported discomfort. The interval between the two EBP was 10 days. Following the second epidural blood patch, symptoms improved for some time, but again, the headache intensity remained 6–7/10. At this stage, the headache exhibited mixed features, with variable intensity regardless of posture, suggesting an overlap of low- and high-pressure phenomena. A repeat MRI of the head revealed stable appearances of the bilateral subdural collections.

As the headaches persisted, maternal medicine MDT suggested investigating further with high-resolution spinal sequences to identify the CSF leak and to try a third targeted higher volume epidural blood patch at the tertiary centre. She was then transferred to a tertiary centre at 21 weeks’ gestation. Meanwhile, her symptoms improved spontaneously, and her pain score went down to 2/10. Hence, the plan for a third targeted epidural blood patch was abandoned.

**Figure 3:**
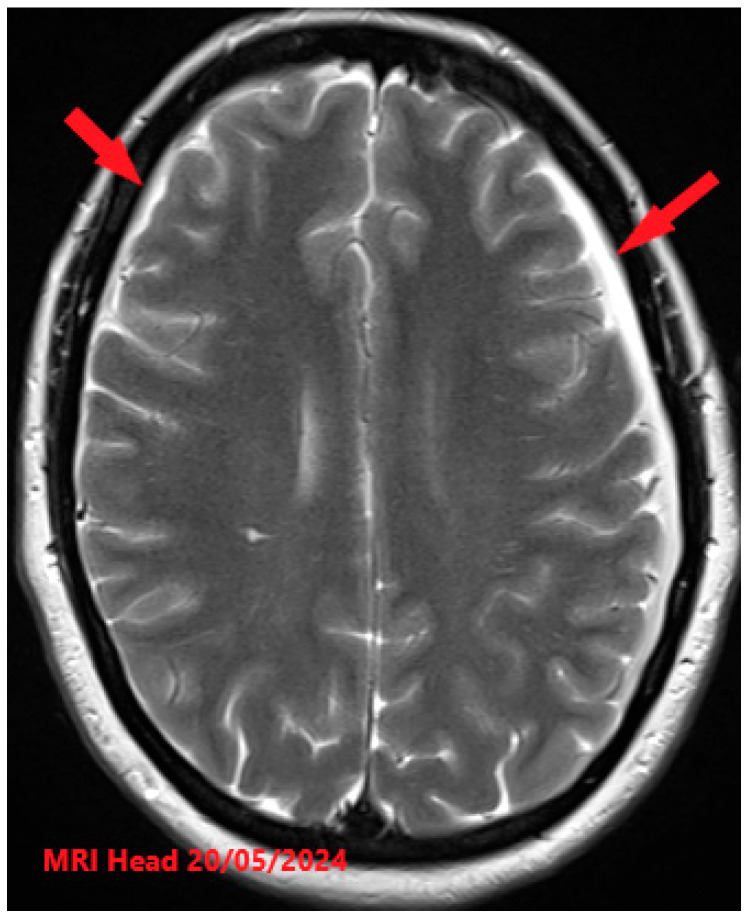
MRI findings (higher resolution) after the treatment showing reduction in subdural collections (red arrows). No definite demonstrable cause of the CSF leak was identified.

**Figure 4:**
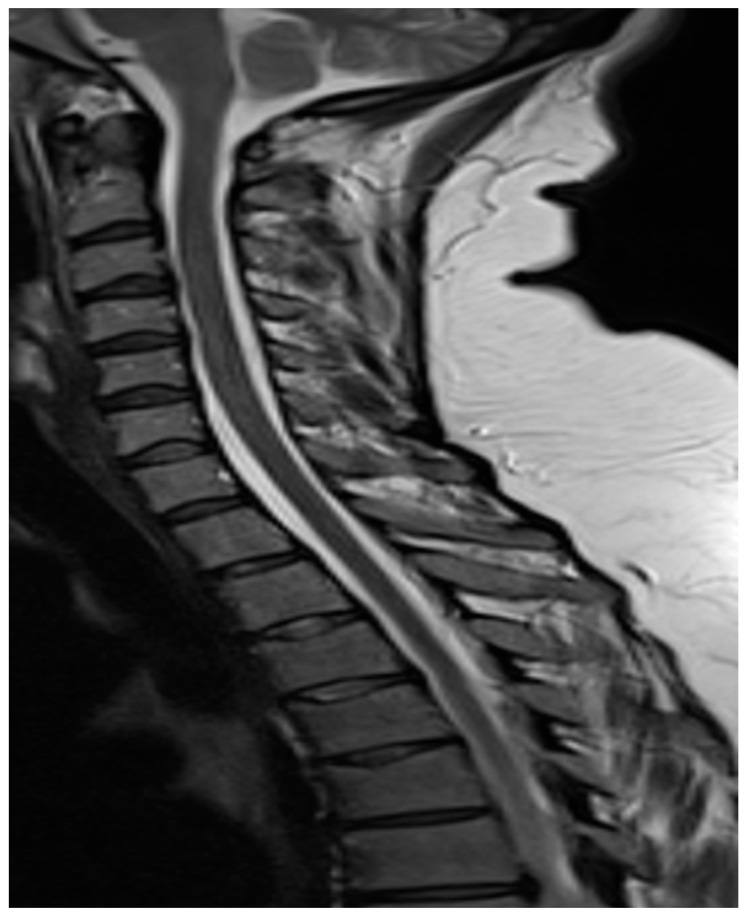
MRI findings (higher resolution) after the treatment showing reduction in lumbar subdural collections. No definite demonstrable cause of the CSF leak was identified.

A follow-up MRI at 24 weeks of pregnancy revealed a reduction in cervical and lumbar spinal CSF collections, with resolution of low-pressure intracranial features (Figure 3 and Figure 4). However, a small volume of interval haemorrhage into the left subdural CSF collection was noted. She was subsequently planned for discharge with advice to maintain a headache diary, ensure adequate hydration, and balance activity and rest periods, including spending 15–20 min in an upright position every two hours. Additional recommendations included avoiding constipation, gradually weaning off analgesics, and arranging a follow-up neurological review at a tertiary centre. As she showed clinical improvement, further imaging was deemed unnecessary by the neurologists. Obstetric management included foetal echocardiography at 24 weeks due to her positive anti-Ro antibodies which are known to cause congenital heart block in the foetus. The foetal echocardiography was normal, and she received fortnightly foetal heart auscultation from 28 weeks. She had serial growth scans at 28, 32, and 36 weeks as she had risk factors for a small-for-gestational age baby. All growth scans were normal.

An anaesthetic review was undertaken, and a caesarean section under general anaesthesia was planned at 38 weeks gestation as neuraxial anaesthesia may worsen the CSF leak and her symptoms. She continued having daily prophylactic dose of low molecular weight heparin (7500 IU dalteparin) during the pregnancy, which was stopped the night before the caesarean section. The caesarean delivery was uneventful, with an estimated blood loss of 550 mL. Post-delivery, she was resumed on low-molecular-weight heparin 6 h post caesarean section and continued for 6 weeks. The postnatal period was uneventful. Her headaches improved significantly, allowing her to gradually return to her daily activities. She was able to manage her symptoms effectively with paracetamol and codeine.

## 3. Discussion

Spontaneous intracranial hypotension (SIH) is a rare but increasingly recognised cause of thunderclap and orthostatic headaches. This case illustrates the complexities of diagnosing and managing SIH in pregnancy, particularly in a patient with multiple comorbidities and overlapping symptomatology. SIH can present with a variety of symptoms, including headache, nausea, vomiting, dizziness, neck stiffness, and in severe cases, even coma. The hallmark feature of SIH is an orthostatic headache, which is typically fronto-occipital, worsens when upright, and improves on recumbency. This is consistent with the patient’s initial presentation to the emergency care facility.

The delay in diagnosis of SIH is not uncommon, as misdiagnosis rates have been reported to exceed 70%, with initial suspicions often including sinusitis, meningitis, or migraine [6]. Median time from presentation to diagnosis is two months. In this case, initial imaging showed chronic maxillary sinusitis, and the patient was treated for presumed acute bacterial sinusitis. Sinus mucosal thickening is frequently an incidental finding on head CT and may mislead clinicians in patients with frontal headaches [7]. The presenting complaint of frontal headache often mimics other conditions, such as migraines, which do not exhibit postural variation, and cervicogenic headaches, where the pain originates in the neck and radiates to the fronto-temporal areas, typically exacerbated by neck movements. It is crucial to rule out more serious conditions, such as meningitis, reversible cerebrovascular vasoconstriction syndrome (RCVS), cerebral venous thrombosis, or subarachnoid haemorrhage. Postural tachycardia syndrome can also add to the diagnostic complexity as it presents with orthostatic headache, but without a CSF leak [8]. It is associated with symptoms such as light-headedness, tremulousness, palpitations, tachycardia, and hypotension, which can be diagnosed by a head-up tilt-table test. According to the International Classification of headache disorders (ICHD-3) [9], either a low opening pressure on lumbar puncture (<6 cm CSF) or typical radiological signs of intracranial hypotension (either direct on spine imaging or indirect on brain imaging) are required for a diagnosis of spontaneous intracranial hypotension. This is in contrast to the diagnostic criteria of SIH proposed by Schievink et al. [10] (Table 1).

Lumbar puncture (LP) may support the diagnosis of SIH, typically showing a low opening pressure (usually < 60 mm H_2_O) and occasionally mild pleocytosis or elevated protein due to meningeal irritation [3]. However, its role is controversial in spontaneous leaks. In this case, LP revealed lymphocytic pleocytosis, which initially misled clinicians toward aseptic meningitis. This is a well-recognised pitfall as the CSF in SIH may show xanthochromia even without subarachnoid haemorrhage, likely due to blood–brain barrier disruption or small subdural bleeds associated with brain sagging [11]. Moreover, traumatic taps may confound interpretation. Importantly, the diagnostic yield of CSF pressure readings is unreliable, as pressure can normalise if the leak is intermittent or compensated, particularly in chronic or subacute cases [12].

Initial management of SIH includes conservative measures with adequate rest, avoidance of prolonged upright position, hydration, caffeine, analgesics, and avoidance of any strenuous activities, bending, or straining. If there is no improvement in symptoms following the conservative management, a blind epidural blood patch is recommended, with reported efficacy of over 70% in non-pregnant populations [13]. Further management is guided by the symptoms. In case of no improvement or some improvement, a second epidural blood patch should be used as followed in our case. The recommended time interval between two epidural blood patches is 2–4 weeks. In more resistant cases, referral to a tertiary neuroscience centre should be considered where facilities for myelography to locate the site and type of CSF leak, as well as targeted epidural blood patch, are available after discussion with the multidisciplinary team. In this case, two blind EBPs provided transient relief. Ultimately, a third targeted EBP was planned but not required due to spontaneous resolution of symptoms, which is not an uncommon occurrence in SIH, where CSF leaks may seal spontaneously over time [14].

In order to contextualise our findings and management decisions, a review of published cases of spontaneous intracranial hypotension (SIH) in pregnancy was undertaken. Previous reports demonstrate a spectrum of severity, timing, and response to therapy, reflecting the diagnostic and therapeutic challenges unique to pregnancy. Table 2 provides a comparative summary of the key case reports and series, including our own, outlining gestational age at presentation, diagnostic findings, management strategies, and maternal–foetal outcomes.

The present case aligns closely with the observations of Ferrante et al. [5], in which symptoms developed during mid-pregnancy and resolved following conservative measures and one or more blind epidural blood patches (EBPs). Compared with series where targeted EBPs were guided by myelographic localisation [16,18,19], our patient illustrates a pragmatic, stepwise approach appropriate when advanced imaging options are restricted during pregnancy. The decision to proceed with caesarean delivery under general anaesthesia was consistent with recommendations to avoid neuraxial techniques while a dural leak remains possible. Across the reviewed literature and in our experience, maternal and neonatal outcomes were uniformly favourable when multidisciplinary coordination and close follow-up were maintained.

Intermediate follow-up is recommended at 10–14 days after an epidural blood patch and at 3–6 weeks after surgical intervention. A late follow-up should be scheduled between 3–6 months to monitor long-term outcomes. If there is no clinical improvement or if initial improvement is followed by a relapse, the patient should be referred back to the multidisciplinary team (MDT) or relevant specialist for further evaluation, which may include repeat imaging or additional intervention. If the patient demonstrates sustained improvement, further specialist or MDT input may not be necessary [20].

Complications of spontaneous intracranial hypotension (SIH) include cerebral venous thrombosis, which may manifest with an abrupt change in the character of the headache or new neurological deficits. Other recognised complications include superficial siderosis, which can present with progressive cerebellar ataxia and sensorineural hearing loss; in such cases, measurement of serum ferritin levels may be informative. Additionally, SIH is associated with an increased risk of subdural collections and subarachnoid haemorrhage [20]. It is therefore essential to accurately recognise the clinical manifestations of SIH and initiate appropriate management, while maintaining a high index of suspicion for potential complications.

The patient’s autoimmune background (Sjögren’s syndrome and anti-Ro positivity) may have predisposed her to meningeal fragility, potentially increasing susceptibility to spontaneous CSF leak, although this association remains speculative and has never been reported in the literature [21]. Furthermore, the presence of a high BMI, prior obstetric complications, and VTE risk added complexity to both diagnosis and management. Delivery via caesarean section under general anaesthesia was appropriate given the risk of dural puncture with neuraxial anaesthesia and the recent EBP, aligning with current recommendations [22].

## 4. Conclusions

Spontaneous intracranial hypotension remains a rare but important cause of severe postural headache in pregnancy, with potential diagnostic and therapeutic challenges due to physiological and imaging constraints. This case highlights the importance of maintaining a high index of suspicion and adopting a multidisciplinary, stepwise approach—beginning with conservative management and escalating to epidural blood patching when clinically indicated. Early recognition and collaboration between obstetric, neurological and anaesthetic teams are key to preventing complications and optimising maternal and foetal outcomes. Although most cases resolve spontaneously or with supportive therapy, careful delivery planning and postpartum follow-up are essential for monitoring for recurrence and guiding future anaesthetic decisions. Further research and pooled case data are needed to establish pregnancy-specific management guidelines and to define the safety and timing of targeted epidural blood patches in this unique population.

This case highlights the importance of considering spontaneous intracranial hypotension (SIH) in pregnant patients presenting with thunderclap headache with an orthostatic component that is usually resistant to regular analgesics, particularly when initial evaluations are non-diagnostic. Increased awareness among clinicians can aid in early diagnosis and prevent unnecessary delays in treatment.

## Figures and Tables

**Table 1:** Diagnostic criteria for SIH as proposed by Schievink et al. [10].

(A) Orthostatic headache
(B) The presence of at least 1 of the following:
(1) Low opening pressure (≤60 mm H_2_O)
(2) Sustained improvement of symptoms after epidural blood patching
(3) Demonstration of an active spinal CSF leak
(4) Cranial MRI changes in intracranial hypotension (e.g., brain sagging or pachymeningeal enhancement)
(C) No recent history of dural puncture
(D) Not attributable to another disorder

**Table 2:** Comparison of management and outcomes of reported SIH cases in pregnancy.

Study/Year	No. of Cases (Pregnant)	Gestational Age at Presentation	Key Clinical Features	Imaging Findings	Management Approach	Anaesthetic/Delivery Considerations	Outcome
Asakura et al., 2001 [15]	1	8 weeks	Sudden severe postural headache	MRI showed diffuse subdural fluid collection, narrowed ambient cistern	Conservative (bed rest)	Unknown	Symptom resolution in 4 weeks
Singh et al., 2009 [16]	1	32 weeks	Postural headache	Typical SIH feature on MRI	Single EBP	SVD at 41 weeks	Symptoms resolved after 3 weeks of conservative management
Reihani et al., 2022 [17]	1	32 weeks	Postural headache	Typical SIH features on MRI	Conservative (caffeine, bed rest)	Planned CS	Symptoms improved after delivery
McGrath et al., 2010 [18]	1 (recurrence)	15 weeks (and 16 weeks)	Intermittent frontal headache	MRI: Cerebellar tonsillar descent with ectopia and flattening of the pontine profile	Single EBP for both pregnancies	SVD at term	Symptoms resolved after EBP
Grange et al., 2016 [19]	1	28 weeks	Postural headache	MRI (Pachymeningeal enhancement, subdural hygroma)	Conservative + targeted EBP	Regional anaesthesia avoided until leak sealed	Full maternal recovery
Ferrante et al., 2020 [5]	5	6-17 weeks	Severe orthostatic headache, 1 complicated by cerebral venous thrombosis	Typical SIH features on MRI	3/5 required 1 EBP, 1/5 required 2 EBP	2 term SVD, 3 caesareans at request of patient; neuraxial avoided in active SIH	Most patients improved after EBP; one case has CVT as complication
Present case (Agrawal et al., 2025)	1	16 weeks	Severe orthostatic headache,	MRI: pachymeningeal enhancement, venous engorgement, extradural CSF, subdural collections	Conservative → Two blind EBPs → Spontaneous improvement before targeted EBP	Elective caesarean under GA; multidisciplinary planning	Complete neurological recovery; healthy neonate

## Data Availability

The original contributions presented in the study are included in the article. Further inquiries can be directed to the corresponding author.
